# Grass carp reovirus VP4 manipulates TOLLIP to degrade STING for inhibition of IFN production

**DOI:** 10.1128/jvi.01583-24

**Published:** 2025-01-14

**Authors:** Yang-Yang Wang, Xue-Li Wang, Zhuo-Cong Li, Can Zhang, Xiao Xu, Bao-Jie Cui, Meng-Ze Tian, Chu-Jing Zhou, Na Xu, Yue Wu, Xiao-Li Yang, Dan-Dan Chen, Long-Feng Lu, Shun Li

**Affiliations:** 1 Institute of Hydrobiology, Chinese Academy of Sciences 53021 , Wuhan, Hubei, China; 2 University of Chinese Academy of Sciences 521953 , Beijing, Beijing, China; 3 TEDA Institute of Biological Sciences and Biotechnology, Nankai University 199184 , Tianjin, Tianjin, China; 4 College of Fisheries and Life Science, Dalian Ocean University 26480 , Dalian, Liaoning, China; 5 Key Laboratory of Aquaculture Disease Control, Ministry of Agriculture, Wuhan, China; 6 Laboratory for Marine Biology and Biotechnology, Qingdao Marine Science and Technology Center, Qingdao, China; 7 State Key Laboratory of Freshwater Ecology and Biotechnology, Institute of Hydrobiology, Wuhan, China; University of Michigan Medical School, Ann Arbor, Michigan, USA

**Keywords:** GCRV, STING, TOLLIP, immune escape, IFN

## Abstract

Although fish possess an effective interferon (IFN) system to defend against viral infection, grass carp reovirus (GCRV) still causes epidemic hemorrhagic disease and tremendous economic loss in grass carp. Therefore, it is necessary to investigate the immune escape strategies employed by GCRV. In this study, we show that the structural protein VP4 of GCRV (encoded by the S6 segment) significantly restricts IFN expression by degrading stimulator of IFN genes (STING) through the autophagy-lysosome-dependent pathway. First, overexpression of VP4 inhibited the expression of IFN induced by GCRV and polyinosinic-polycytidylic acid (poly I:C) at both the promoter and mRNA levels. Second, VP4 was found to associate with STING, and the N-terminal transmembrane domain is essential for this interaction. Additionally, VP4 dramatically blocked STING-induced IFN expression and weakened its antiviral capacity. Further mechanistic studies revealed that VP4 degrades STING via the autophagy-lysosome pathway in a dose-dependent manner. Interestingly, toll-interacting protein (TOLLIP), a selective autophagy receptor, was found to interact with VP4 and reduce VP4-mediated STING degradation after *tollip* knockdown. Finally, overexpression of VP4 facilitated GCRV proliferation, while its depletion had the opposite effect. These findings indicate that GCRV VP4 recruits TOLLIP to degrade STING and achieve immune escape. This enhances our comprehension of aquatic virus pathogenesis.

**IMPORTANCE:**

Upon virus invasion, fish cells employ a multitude of strategies to defend against infection. Consequently, viruses have evolved a plethora of tactics to evade host antiviral mechanisms. To date, fewer studies have been conducted on the immune evasion mechanism of grass carp reovirus (GCRV). In this study, we demonstrate that VP4 of GCRV-873 inhibits interferon expression by interacting with stimulator of IFN gene and degrading it in an autophagy-lysosome-dependent manner through the manipulation of the selective autophagy receptor toll-interacting protein. The findings of this study contribute to our understanding of the novel evasion mechanisms of GCRV and widen our knowledge of the virus-host interactions in lower vertebrates.

## INTRODUCTION

Grass carp reovirus (GCRV) is a highly virulent virus that causes epidemics and outbreaks of hemorrhagic disease in grass carp, resulting in mass mortality (1). It is a double-stranded RNA (dsRNA) virus that belongs to the genus *Aquareovirus* in the family *Reoviridae* (2). The known GCRV strains can be divided into three subtypes (I, II, and III) based on gene sequences (2). The three representatives of GCRV are GCRV-873 (GCRV-I), GCRV-HZ08 (GCRV-II), and GCRV-104 (GCRV-III) (2). In addition, three types of GCRV exhibit significant differences in nucleotide sequences, viral-encoded protein structures, and pathogenicity in both grass carp and (*Ctenopharyngodon idellus*) kidney (CIK) cells (3). Notably, GCRV-873 was the first aquareovirus to be characterized and sequenced, and it caused obvious cytopathic effects (CPEs) in CIK cells (4). Its genome is composed of 11 dsRNA segments (named S1–S11) and encodes seven structural proteins and five non-structural proteins (5). Segment 6 of GCRV-873 is predicted to encode the capsid protein VP4, which has a predicted molecular weight of 68.58 kDa and is a putative cofactor/nucleoside triphosphate phosphohydrolase (6). While many studies have been conducted on GCRV, mainly focused on investigating the virus itself, the pathogenesis and immune responses of GCRV in grass carp remain to be elucidated (7, 8). Only a few studies have reported on the immune evasion strategies employed by GCRV. For instance, NS38 and NS80 of GCRV-873 were found to inhibit interferon (IFN) production by targeting the retinoic-acid inducible gene I (RIG-I)-like receptor (RLRs) pathway (9). Moreover, GCRV-873 was used as a target for disease-resistant breeding and virus-host interaction in early studies (3, 10, 11). Therefore, it is important to explore additional strategies to counteract the host IFN response utilized by GCRV-873.

IFN is the primary defense against viral invasion in mammals and fish (12, 13). During viral infection, host pattern recognition receptors recognize viral nucleic acids and trigger the activation of a signaling cascade to initiate antiviral immune response (14). The RLR signaling pathway plays a critical role in activating IFN expression and comprises three members: retinoic acid-inducible gene-I (RIG-I), melanoma differentiation-associated gene 5 (MDA5), and laboratory of genetic and physiological 2 (15, 16). Upon binding to viral RNA, RIG-I or MDA5 recruits the downstream junction mitochondrial antiviral signaling protein (MAVS, also known as VISA, IPS-1, or Cardif), which then transmits the signal to the downstream stimulator of the IFN gene (STING, also known as mediator of IRF3 activation (MITA), endoplasmic reticulum (ER) IFN stimulator (ERIS), or plasma membrane tetraspanner (MPYS)) and TANK-binding kinase 1 (TBK1) (17, 18). Upon phosphorylation by TBK1, IFN regulatory factor 3 (IRF3) and IRF7 are activated and translocate to the nucleus to trigger IFN transcription (19). Similar to mammals, fish also have a conserved RLR-triggered IFN response mediated by the MAVS-STING-TBK1-IRF3 pathway (20, 21).

STING is a crucial molecule in the RLR pathway. It functions as a scaffold protein that interacts with MAVS and promotes TBK1-mediated phosphorylation of IRF3/7, leading to IFN induction (17, 22). STING homologs have been identified in several fish species, including crucian carp (*Carassius auratus* L.), zebrafish (*Danio rerio*), and fathead minnow (*Pimephales promelas*), and have been functionally characterized (23–25). As STING plays an important role in inducing antiviral immune responses, it has become a hot target for many viruses attempting to evade the immune system. For instance, open reading frame (ORF)10 of the severe acute respiratory syndrome coronavirus 2 (SARS-CoV-2) impairs the cyclic GMP-AMP synthase (cGAS)-STING signaling by blocking the interaction between STING and TBK1, thereby suppressing IFN production (26). The nonstructural protein NS4B of the hepatitis C virus (HCV) interacts with STING to inhibit IFN signaling (27). MGF-505–7R of the African swine fever virus (ASFV) degrades STING through the autophagy pathway (28). The kinase-like protein of cyprinid herpesvirus 2 degrades MITA in an autophagy-lysosome-dependent manner to suppress IFN production (29). Although there have been few studies on STING and fish virus, it is unclear whether the immune escape of GCRV-873 is associated with STING.

To data, there are limited studies on the immune evasion mechanisms of GCRV-873. In this study, we found that VP4 of GCRV-873 interacts with STING and degrades it in an autophagy-lysosome-dependent manner by manipulating the selective autophagy receptor toll-interacting protein (TOLLIP), resulting in the inhibition of IFN production and the facilitation of GCRV proliferation. These data reveal the novel evasion mechanism used by GCRV-873 of targeting STING and expand our knowledge of the virus-host associations in lower vertebrates.

## RESULTS

### VP4 blocks GCRV and polyinosinic-polycytidylic acid (poly I:C)-induced IFN activation

**Fig 1:**
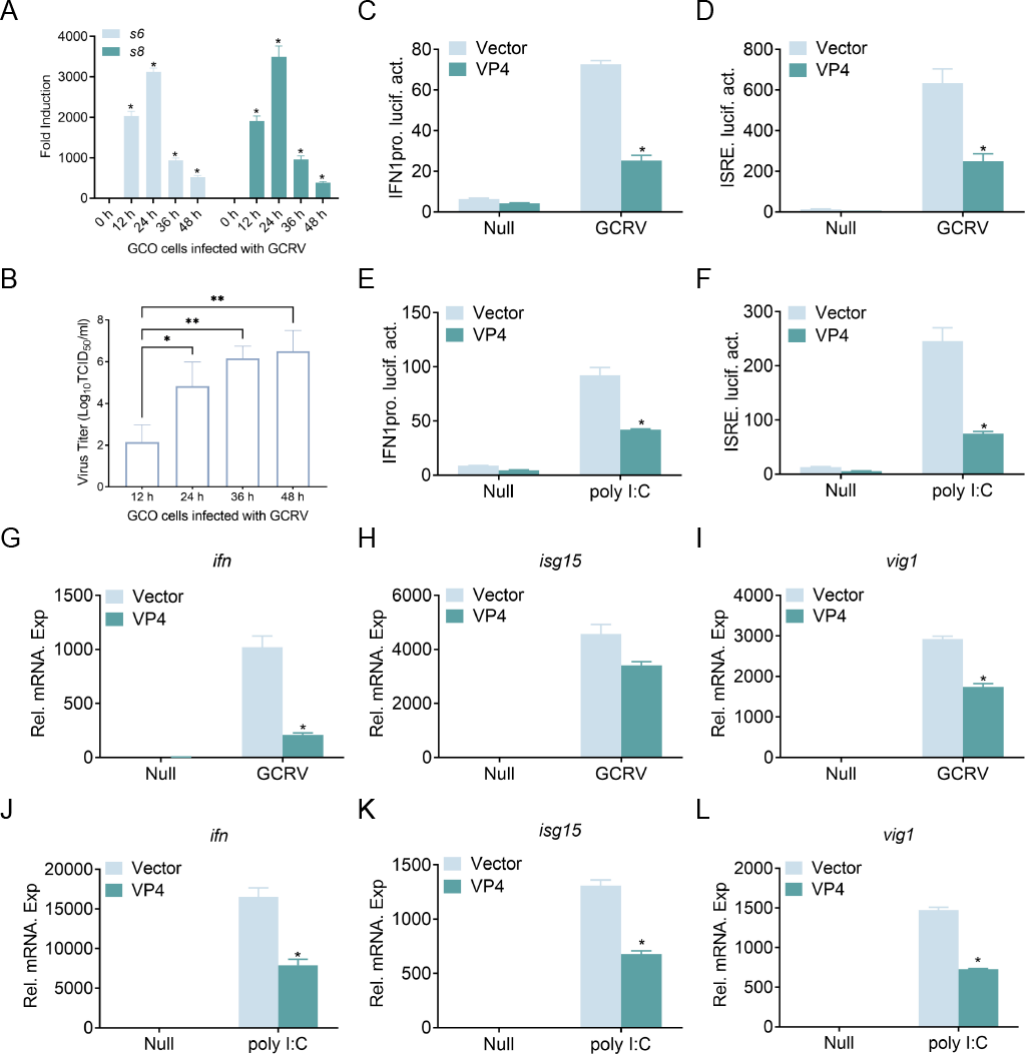
VP4 blocks GCRV/poly I:C-induced IFN expression. (**A and B**) Quantitative PCR (qPCR) detection of the transcriptional levels of *s6* and *s8* and viral titer measurement on GCRV stimulation. GCO cells were seeded on six-well plates overnight and stimulated with GCRV (multiplicity of infection [MOI] = 10). Total RNAs were extracted at time points 0, 12, 24, 36, and 48 h for further qPCR assays, and the supernatant is collected for viral titer measurement. (**C–F**) The activation of IFN1pro/ISRE-Luc induced by GCRV or poly I:C is suppressed by the overexpression VP4. GCO cells were seeded in 24-well plates overnight and transfected with 250 ng IFN1pro-Luc or ISRE-Luc and 50 ng pRL-TK, together with 250 ng VP4-Flag or control vector. After 24 h, cells were untreated (Null) and infected with GCRV (MOI = 100) or transfected with poly I:C (1 mg/mL), respectively. Luciferase activities were monitored 24 h after stimulation. (**G–L**) The transcription of *ifn*, *isg15*, and *vig1* induced by GCRV or poly I:C is inhibited by the overexpression of VP4 in GCO cells. GCO cells were seeded in six-well plates overnight and then transfected with the indicated plasmids (2 µg each). At 24 h, the cells were transfected with poly I:C or infected with GCRV (MOI = 10) for 24 h, and total RNAs were extracted to examine the mRNA levels of cellular *ifn*, *isg15*, and *vig1. β-actin* was utilized as an internal control for normalization, and the relative expression is presented as fold induction relative to the expression level in control cells (set to 1). Data were expressed as mean ± SEM, *n* = 3. Asterisks indicate significant differences from control (*P < 0.05). All experiments were repeated at least three times with similar results.

As one virus may possess multiple strategies to escape host immune system, other immune escape mechanisms of GCRV-873 need to be clarified. Here, to further comprehend the other strategies used by GCRV to combat the host, other plasmids of GCRV-873 segments were employed in luciferase experiments *in vitro*, and the *s6*-encoded protein (VP4) exhibited the potential to suppress host IFN activation. Upon infected with GCRV, the transcripts of viral *s6* and *s8* gene were highly expressed, with the highest expression of *s6* and *s8* reaching 3,123 and 3,494-fold at 24 h post-infection (PI), respectively (Fig. 1A). Moreover, the viral titer of GCRV in grass carp ovary (GCO) cells gradually increased from 12 to 48 h, with the highest viral titers observed at 48 h PI and reaching 10^6.50^ TCID_50_/mL (Fig. 1B). These data indicate that GCRV efficiently replicates in GCO. Although grass carp type I IFNs contain four members (IFN1–IFN4), only IFN1 could be activated by poly I:C, which is a mimic of viral RNA (30). Thus, the IFN1 promoter (IFN1pro) activity was detected in the following study. As shown in Fig. 1C and E, GCRV infection or poly I:C stimulation significantly induced IFN1pro activities, whereas these inductions were remarkably impeded by overexpression of VP4. IFN-stimulated regulatory element (ISRE) is a transcription factor-binding motif found in the promoter regions of IFNs and IFN-stimulated genes (ISGs). Consistently, GCRV or poly I:C-triggered ISRE activities were also dampened in overexpressed-VP4 cells (Fig. 1D and F). Moreover, the mRNAs of *ifn1*, *isg15*, and *vig1* in GCO cells were upregulated by GCRV or poly I:C stimulation, and a noticeable downregulation was observed in the VP4 group (Fig. 1G through L). Collectively, these data suggest that GCRV VP4 serves as a negative regulator to inhibit host IFN expression.

### VP4 associates with the transmembrane of STING and locates in the cytoplasm

**Fig 2:**
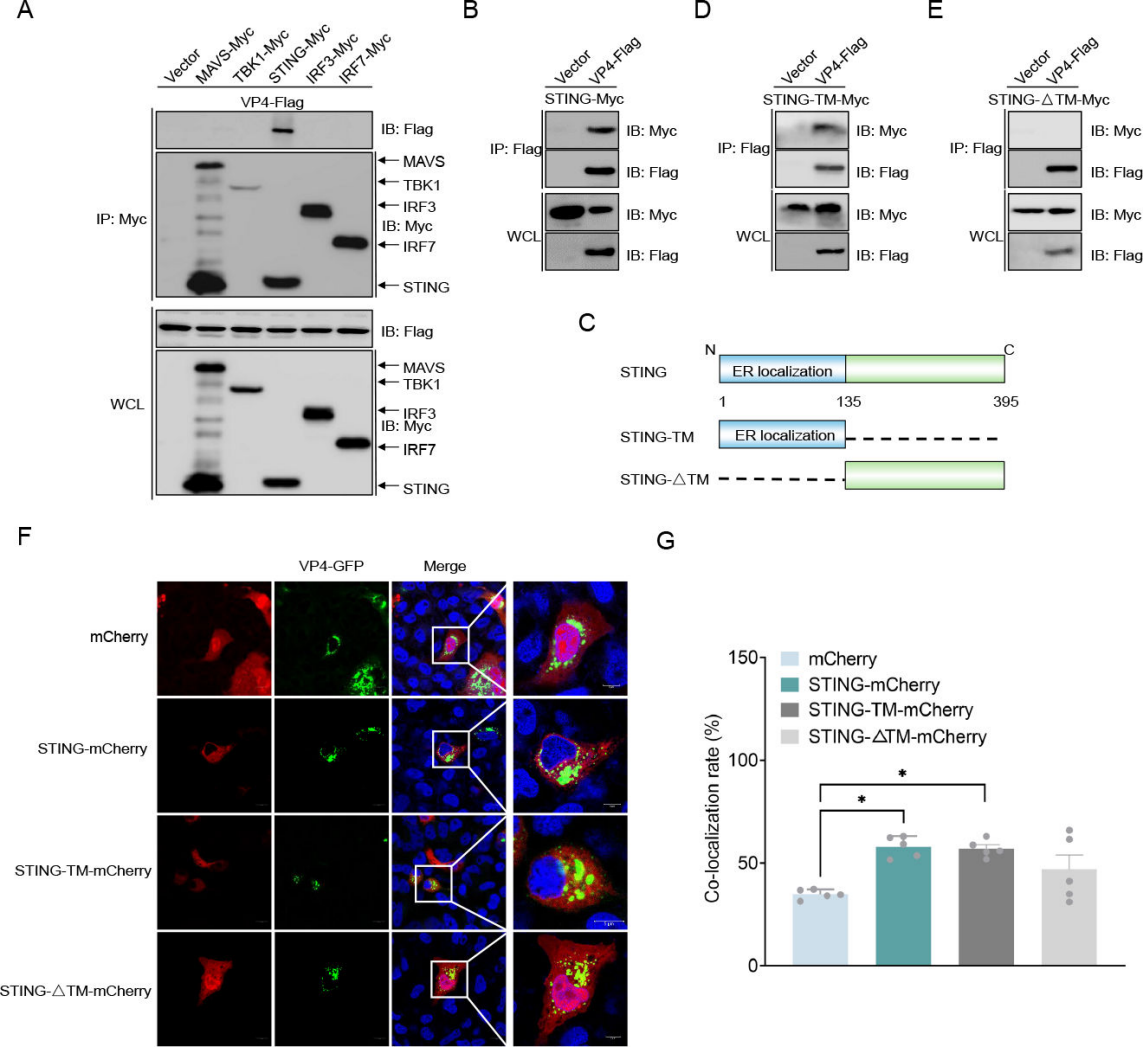
VP4 interacts with the TM structural domain of STING. (**A and B**) VP4 associated with STING. GCO cells seeded in 10 cm^2^ dishes were transfected with the indicated plasmids (5 µg each). After 24 h, cell lysates were immunoprecipitated with anti-Myc (**A**) or anti-Flag (**B**) affinity gel. The immunoprecipitates and whole cell lysates (WCLs) were detected by immunoblotting (IB) with anti-Flag and anti-Myc Abs, respectively. (**C**) Schematic representation of deletion mutants of STING. There are two mutants of STING: STING-TM, containing the N-terminal 135 aa; and STING-ΔTM, containing the C-terminal 260 aa. (**D and E**) VP4 interacts with STING through its TM domain. GCO cells seeded in 10 cm^2^ dishes were transfected with the indicated plasmids (5 µg each). After 24 h, cell lysates were immunoprecipitated with anti-Flag affinity gel. The immunoprecipitates and WCLs were detected by IB with anti-Flag and anti-Myc Abs, respectively. (**F and G**) VP4 is mainly distributed in the cytoplasm and partially overlaps with STING/STING-TM red signaling. GCO cells were plated onto coverslips in six-well plates and co-transfected with the indicated plasmids (1 µg each). After 24 h, the cells were fixed and observed by confocal microscopy. Green signals represent overexpressed VP4-GFP, red signals represent overexpressed STING-mCherry, STING-TM-mCherry, STING-ΔTM-mCherry, or empty vector, and blue staining indicates the nucleus region (**F**) (original magnification 63×; oil immersion objective). Scale bar, 20 µm and 5 µm. The overlap coefficient is expressed as the co-localization rate, and the number of measurements analyzed to determine the confocal rate was five (**G**). All experiments were repeated at least three times with similar results.

Since the fish RLR signaling pathway plays a central role in the induction of IFN expression, it was necessary to investigate the relationships between VP4 and RLRs (24). First, VP4-Flag and Myc-tagged RLR molecules (MAVS/TBK1/STING/IRF3/IRF7) were co-expressed in GCO cells for co-immunoprecipitation (Co-IP) assay. The results showed that only the anti-Myc Ab-immunoprecipitated STING bound to the Flag-tagged VP4, and the reverse IP assay showed that the anti-Flag Ab-immunoprecipitated VP4 was also bound to the Myc-tagged STING, suggesting that VP4 associates with STING (Fig. 2A and B). To identify which domain of STING interacts with VP4, we constructed the STING deletion mutants: STING-transmembrane (TM), only the N-terminal region, and STING-ΔTM, lacking the TM region (Fig. 2C). And the Co-IP assays showed that VP4 binds to STING-TM but not to STING-ΔTM, indicating that the TM region of STING is essential for VP4 interaction with STING (Fig. 2D and E). Next, the subcellular localization of VP4 with STING, STING-TM, and STING-ΔTM was analyzed in GCO cells. Confocal microscopy analysis and co-localization rate revealed that the GFP signals enhanced by VP4 were mainly distributed in the cytoplasm as patchy aggregates and partially overlapped with the red signals of STING and STING-TM (Fig. 2F and G). These data suggest that VP4 binds to STING and locates to the cytoplasm, and the N-terminal TM of STING is required for this interaction.

### VP4 blocks STING-induced cellular antiviral responses

**Fig 3:**
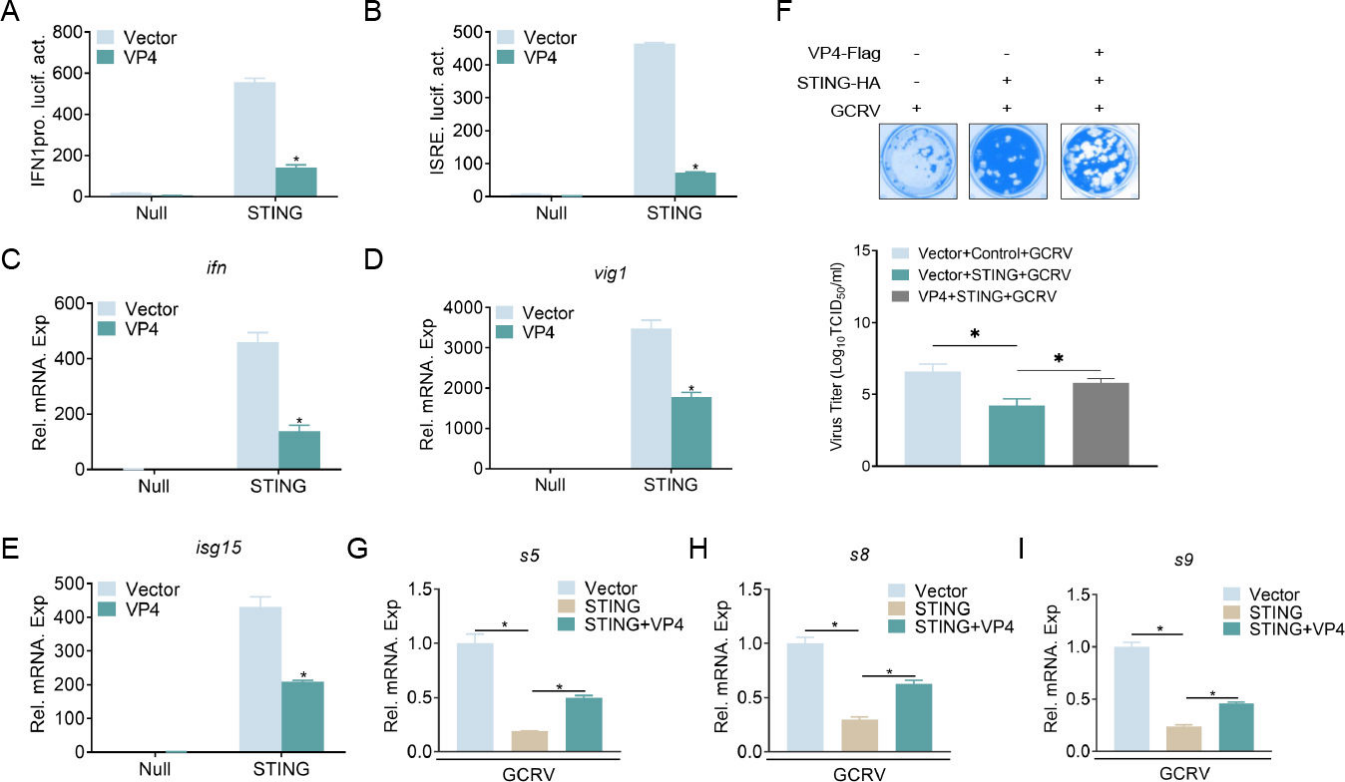
VP4 inhibits STING-mediated cellular antiviral responses. (**A and B**) Overexpression of VP4 blocks STING-mediated IFN1pro/ISRE activation. GCO cells were seeded in 24-well plates overnight and transfected with 250 ng IFN1pro-Luc or ISRE-Luc, 250 ng STING-pcDNA3.1(+) and 50 ng pRL-TK, together with 250 ng VP4-Flag or empty vector. After 24 h, cells were lysed for luciferase activity detection. The promoter activity is presented as relative light units (RLUs) normalized to *Renilla* luciferase activity. (**C–E**) Overexpression of VP4 inhibits the expression of *ifn*, *vig1*, and *isg15* induced by STING. GCO cells were seeded in six-well plates overnight and then transfected with 1 µg STING-HA plus 1 µg VP4-Flag or empty vector. After 24 h, total RNAs were extracted to examine the mRNA levels of cellular *ifn* (**C**), *vig1* (**D**), and *isg15* (**E**). The transcriptional levels were normalized to those of the *β-actin* gene and represented as fold induction relative to the control cells, which were set to 1. (**F**) Overexpression of VP4 counteracts the antiviral effect of STING. GCO cells were seeded in 24-well plates overnight and then transfected with 0.5 µg STING-HA plus 0.5 µg VP4-Flag or empty vector. After 24 h, cells were infected with GCRV (multiplicity of infection [MOI] = 0.1) for 48 h. Subsequently, the cells were fixed with 4% polyformaldehyde (PFA) and stained with 1% crystal violet. Culture supernatants were collected from GCRV-infected cells, and the viral titer was measured using the Reed and Muench method. (**G–I**) Overexpression of STING inhibits viral gene expression. GCO cells were seeded in six-well plates overnight and subsequently transfected with indicated plasmids. At 24 h, cells were infected with GCRV (MOI = 10). After 24 h, total RNAs were extracted to examine the mRNA levels of *s5* (**G**), *s8* (**H**), and *s9* (**I**). The relative transcriptional levels were normalized to the transcriptional level of the *ꞵ-actin* gene and represented as fold induction relative to the transcriptional level in the control cells, which was set to 1. Data were expressed as mean ± SEM, *n* = 3. Asterisks indicate significant differences from control values (*P < 0.05). All experiments were repeated at least three times with similar results.

STING is an important antiviral protein that plays a crucial role in responding to RNA and double-stranded DNA (dsDNA) viruses (24). It is necessary to evaluate whether VP4 affects STING-mediated cellular antiviral responses since VP4 interacts with STING. First, the effects of VP4 on STING-induced IFN production were detected. The results showed that overexpression of VP4 significantly inhibited STING-induced IFN1pro and ISRE activities (Fig. 3A and B). At the mRNA level, the expression of *ifn* and other ISGs induced by STING was attenuated by the overexpression of VP4 (Fig. 3C through E). Subsequently, the impact of VP4 on the antiviral function of STING was investigated. As shown in Fig. 3F, the overexpression of STING protected cells from viral infection and reduced viral titers by 239-fold (from 10^6.60^ to 10^4.22^ TCID_50_/mL at 48 h PI). In contrast, overexpressed VP4 hindered STING’s antiviral function and increased viral titers by 38-fold (from 10^4.22^ to 10^5.80^ TCID_50_/mL at 48 h PI). Additionally, STING overexpression significantly suppressed viral genes expression at the mRNA level, which was reversed by VP4 overexpression (Fig. 3H and I). Collectively, these results suggest that VP4 inhibits STING-mediated cellular antiviral responses.

### VP4 degrades STING at the protein level and displays a dose-dependent manner

**Fig 4:**
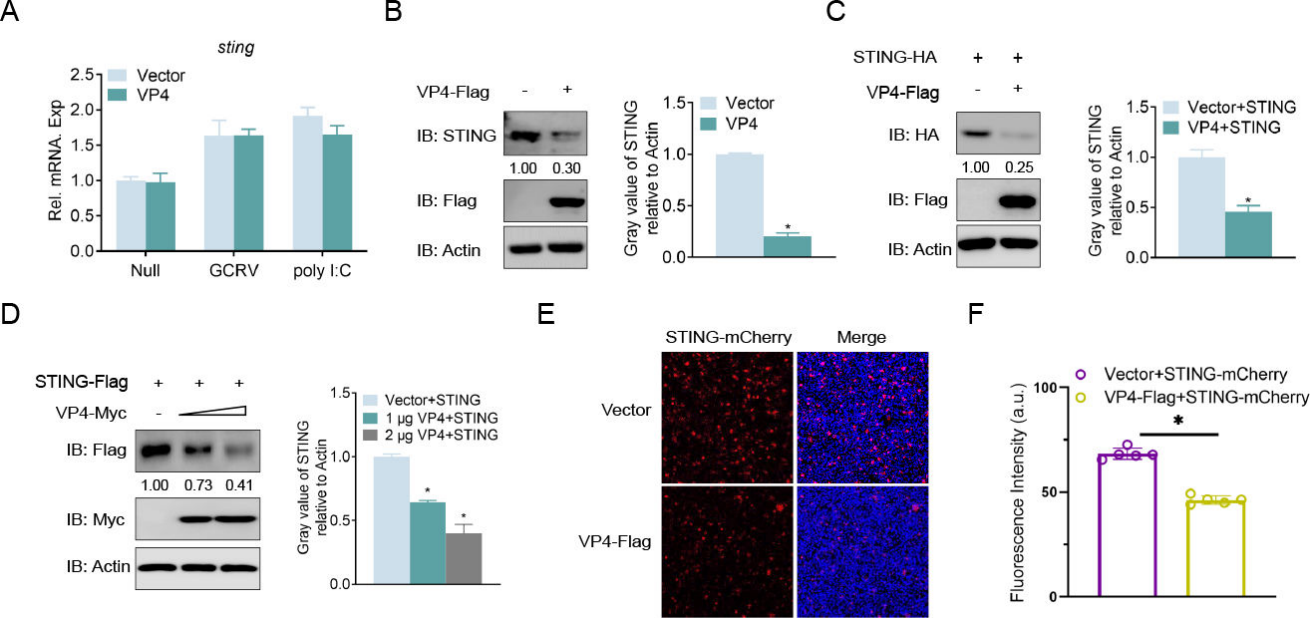
VP4 degrades STING at the protein level. (**A**) Overexpression of VP4 does not affect the transcription of *sting*. GCO cells were seeded in six-well plates overnight and transfected with 2 µg VP4-Flag or empty vector. At 24 h, cells were respectively untreated (Null) and infected with GCRV (multiplicity of infection [MOI] = 10) or transfected with poly I:C (1 mg/mL). After 24 h, total RNAs were extracted to examine the mRNA levels of cellular *sting*. (**B–D**) Overexpression of VP4 degrades STING in a dose-dependent manner. GCO cells were seeded in six-well plates overnight and transfected with (2 µg or 1 µg) of VP4-Flag or empty vector and 1 µg STING-HA (**C and D**) for 24 h. The cell lysates were subjected to immunoblotting (IB) with anti-STING or anti-HA, anti-Flag, and anti-*β-actin* Abs. The grayscale of the three experimental replicates of Fig. 4B through D was statistically analyzed separately. (**E and F**) Overexpression of VP4 degrades STING at the fluorescent level. GCO cells were plated onto coverslips in six-well plates and co-transfected with 1 µg or 2 µg VP4-Flag or empty vector and 1 µg STING-mCherry. After 24 h, the cells were fixed and observed by confocal microscopy. Red signals represent overexpressed STING-mCherry, and blue staining indicates the nucleus region (original magnification ×20 and non-immersion objective) (**E**). Fluorescence intensity of STING-mCherry was statistically analyzed (**F**). All experiments were repeated at least three times with similar results.

The above results indicated that VP4 blocked STING-induced cellular antiviral responses. Therefore, it is essential to investigate the relationship between VP4 and STING. First, we monitored the effect of overexpressed VP4 on STING at mRNA level. The results showed that overexpression of VP4 did not affect *sting* transcripts during GCRV infection or poly I:C stimulation (Fig. 4A). Interestingly, overexpression of VP4 led to significant degradation of endogenous STING and STING-HA at the protein level (Fig. 4B and C). Additionally, the abundance of exogenous STING-HA was also decreased in the VP4-overexpressed group in a dose-dependent manner (Fig. 4D). Furthermore, overexpression of VP4 resulted in a significant reduction in the fluorescence intensity of STING-mCherry (Fig. 4E and F). Taken together, these data suggest that VP4 degrades both endogenous and exogenous STING at the protein level.

### VP4 degrades STING via the autophagy-lysosome pathway

**Fig 5:**
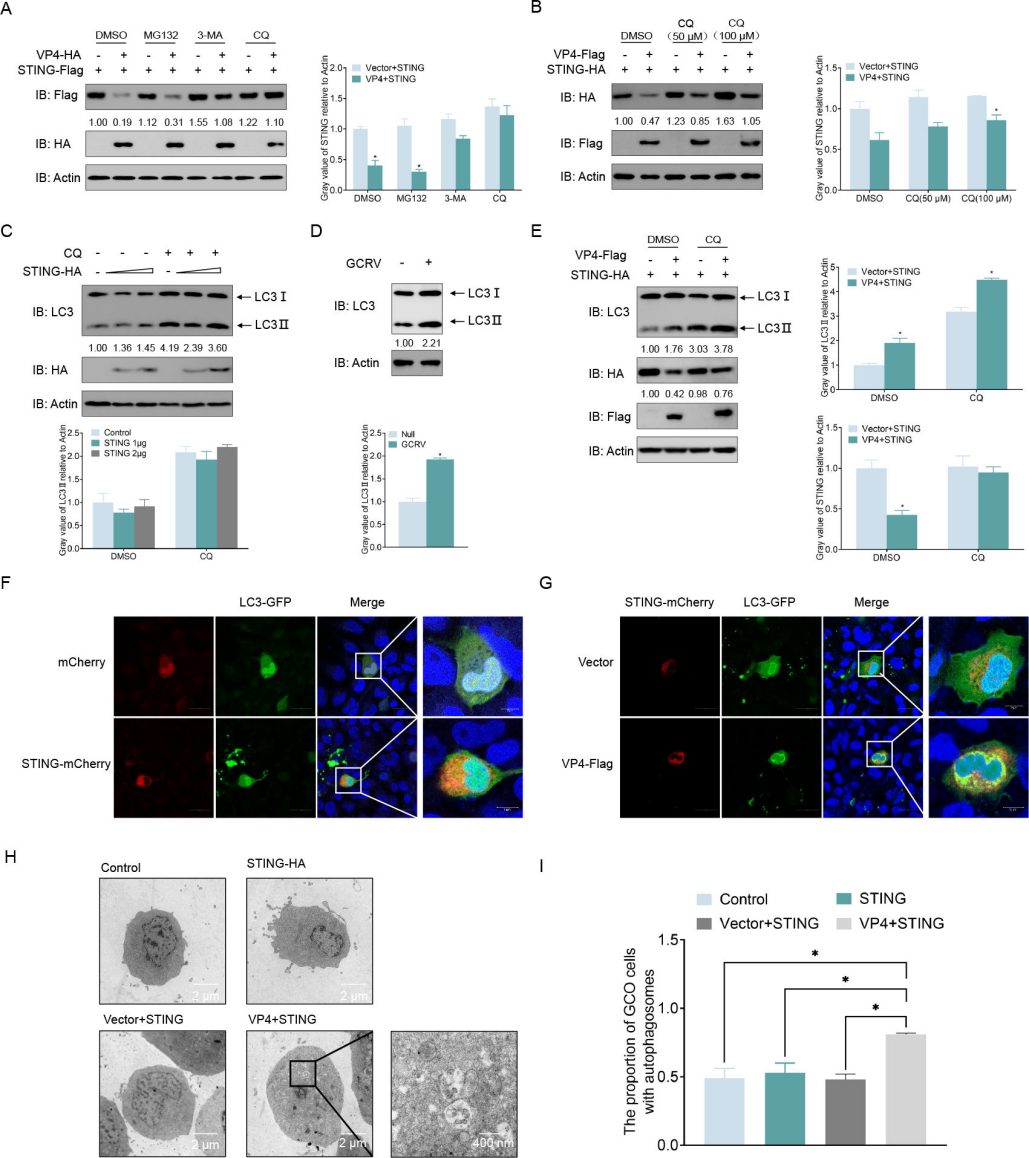
VP4 degrades STING via an autophagy-dependent lysosomal pathway. (**A**) Effects of inhibitors on VP4-mediated degradation of STING. GCO cells were seeded in six-well plates overnight and transfected with the indicated plasmids (1 µg each). At 18 h, the cells were treated with dimethyl sulfoxide (DMSO), MG132 (20 µM), 3-MA (2 mM), Baf-A1 (100 nM), or CQ (100 µM) for 6–8 h. The cells were then harvested for immunoblotting (IB) with anti-HA, anti-Flag, and anti-*β-actin* Abs. (**B**) VP4-induced STING degradation is rescued by CQ in a dose-dependent manner. GCO cells were seeded in six-well plates overnight and co-transfected with the indicated plasmids (1 µg each). At 18 h, the cells were treated with CQ (50 or 100 µM) for 6–8 h. Then, the cells were harvested for IB with anti-HA, anti-Flag, and anti-*β-actin* Abs. (**C**) Overexpression of STING could not induce autophagy. GCO cells were seeded in six-well plates and transfected with 2 µg STING-HA. At 18 h, the cells were treated with DMSO or CQ (50 or 100 µM) for 6–8 h. Then, the cells were harvested for IB with anti-LC3, anti-HA, and anti-*β-actin* Abs. (**D**) Infection with GCRV induces autophagy. GCO cells were seeded in six-well plates and infected with GCRV (multiplicity of infection [MOI] = 10) for 36 h. Then, the cells were harvested for IB with anti-LC3 and anti-*β-actin* Abs. (**E**) Co-transfection of VP4 and STING induces autophagy. GCO cells were seeded in six-well plates overnight and co-transfected with the indicated plasmids (1 µg each). At 18 h, the cells were treated with DMSO and CQ (50 µM) for 6–8 h. The cell lysates were subjected to IB with the anti-LC3, anti-HA, anti-Flag, and anti-*β-actin* Abs, respectively. (**F and G**) Co-expression of VP4 with STING results in the aggregation of LC3-GFP. GCO cells were plated onto coverslips in six-well plates and co-transfected with the indicated plasmids (1 µg each). After 24 h, the cells were fixed and observed by confocal microscopy. Red signals represent STING-mCherry, green signals represent overexpressed LC3-GFP, and blue staining indicates the nucleus region (original magnification 63×; oil immersion objective). Scale bar, 5 µm. (**H and I**) Autophagosome-like structures detection by TEM. GCO cells were seeded in six-well plates overnight and transfected with indicated plasmids (1 µg or 2 µg) for 24 h. The cells were then analyzed by TEM, an enlarged section indicated autophagic vesicles. The proportion of 50 randomly selected cells containing autophagosome-like structures was counted and statistically analyzed. All experiments were repeated at least three times with similar results.

Protein degradation in organisms regulates protein function mainly through the ubiquitin-proteasome pathway and the autophagy-lysosome pathway. To determine the exact mechanism of STING degradation regulated by VP4, several inhibitors were used, including MG132, 3-methyladenine (3-MA), and chloroquine (CQ), which block the ubiquitin-proteasome, autophagosome, and lysosomal pathways, respectively. As shown in Fig. 5A, MG132 treatments did not affect the degradation of STING induced by VP4, whereas the autophagy pathway inhibitors 3-MA and CQ significantly blocked STING degradation. Furthermore, increasing doses of CQ gradually restored the levels of STING protein (Fig. 5B). To confirm that VP4 degrades STING via the autophagy-lysosome pathway, the changes in the amount of LC3-II (which is a reliable indicator of autophagy that can be used to monitor autophagy processes) in both cases of overexpression of STING alone and co-expression of VP4 and STING were examined. Interestingly, there was no change in the amount of LC3-II when STING was overexpressed alone, whereas GCRV infection and co-expression of VP4 and STING resulted in an increase in the expression of LC3-II (Fig. 5C through E). The phenomenon of LC3-GFP puncta formation is regarded as an indicator of increased autophagy levels. Confocal microscopy analysis showed that LC3-GFP punctate was rarely observed when STING was overexpressed alone, whereas a strong green signal of LC3 punctate aggregates appeared when VP4 and STING were co-expressed (Fig. 5F and G). Additionally, transmission electron microscopy (TEM) was used to detect autophagosome-like vesicles to confirm autophagosome formation. As shown in Fig. 5H and I, more autophagosome-like vesicles were observed in cells co-overexpressing VP4 and STING compared to when STING was overexpressed alone. These data show that VP4 degrades STING through the autophagy-lysosome pathway.

### TOLLIP is essential for VP4-mediated autophagic degradation of STING

**Fig 6:**
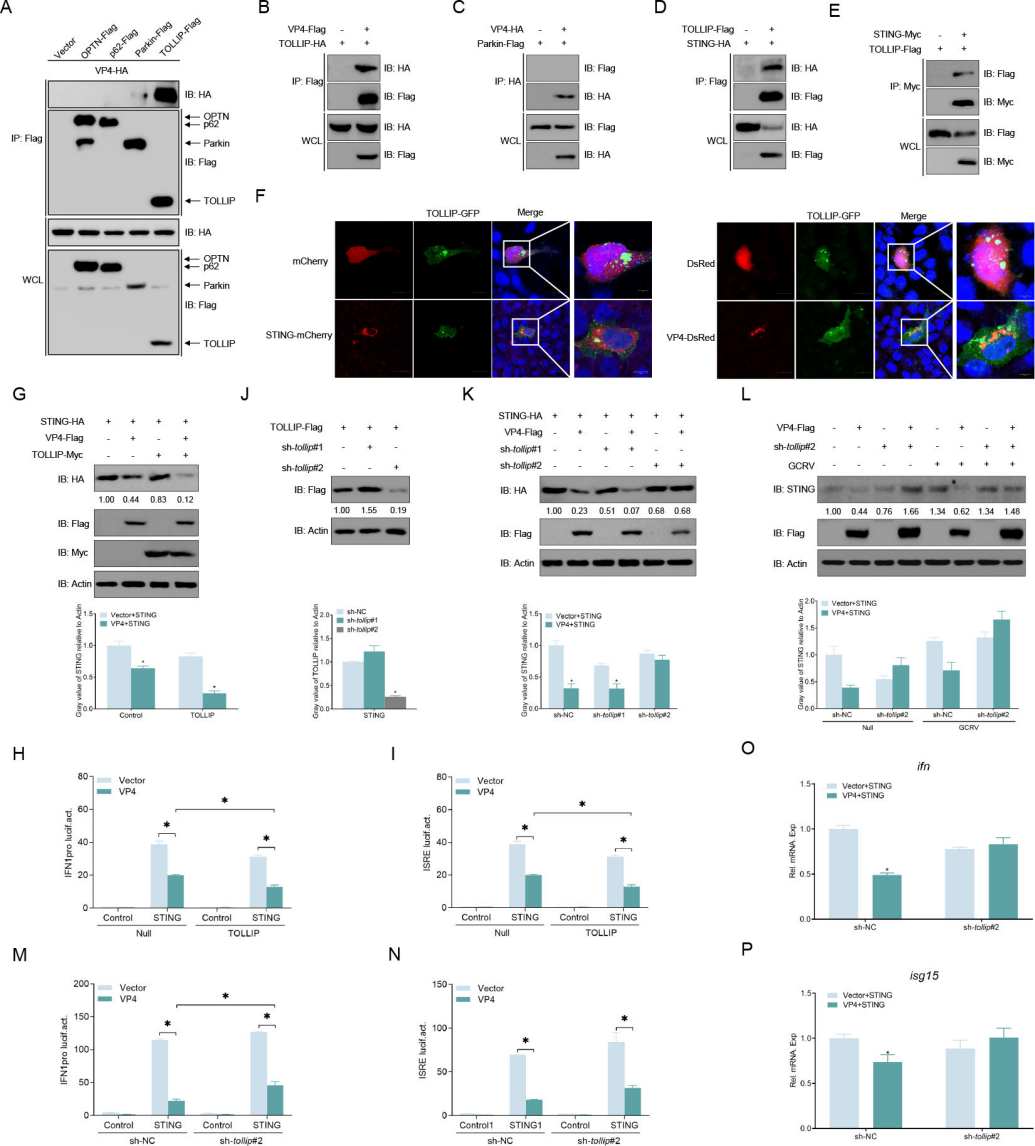
TOLLIP is crucial for VP4-induced degradation of STING. (**A–E**) Both VP4 and STING interact with TOLLIP. GCO cells seeded in 10 cm^2^ dishes were transfected with the indicated plasmids (5 µg each). After 24 h, cell lysates were immunoprecipitated with anti-Flag (A, B, and D), anti-Myc (**E**), or anti-HA (**C**) affinity gel. The immunoprecipitates and whole cell lysates (WCLs) were detected by immunoblotting (IB) with anti-HA, anti-Flag, and anti-Myc Abs, respectively. (**F**) Both VP4 and STING colocalizes with TOLLIP. GCO cells were plated onto coverslips in six-well plates and co-transfected with 1 µg TOLLIP-EGFP and 1 µg empty vector, STING-mCherry or VP4-DsRed, respectively. After 24 h, the cells were fixed and subjected to confocal microscopy analysis. Green signals represent overexpressed TOLLIP, red signals represent overexpressed STING or VP4, and blue staining indicates the nucleus region (original magnification 63×; oil immersion objective). Scale bar, 5 µm. (**G**) TOLLIP promotes the degradation of STING by VP4. GCO cells were seeded in six-well plates overnight and co-transfected with the indicated plasmids. At 24 h, the cell lysates were subjected to IB with the anti-HA, anti-Flag, anti-Myc, and anti-*β-actin* Abs, respectively. (**J**) Validation of knock-down efficiency of TOLLIP. GCO cells seeded in six-well plates were transfected with 1 µg TOLLIP-Flag and 1 µg sh-*tollip*#1-PLKO.1, sh-*tollip*#2-PLKO.1, or control (sh-NC). At 24 h, the cell lysates were subjected to IB with the anti-Flag and anti-*β-actin* Abs. (**K**) Knockdown of *tollip* by sh-*tollip*#2 effectively rescues the degradation of exogenous STING by VP4. GCO cells were seeded in six-well plates overnight and co-transfected with the indicated plasmids. At 24 h, the cell lysates were subjected to IB with the anti-HA, anti-Flag, and anti-*β-actin* Abs, respectively. (**L**) Knockdown of *tollip* rescues degradation of endogenous STING by VP4. GCO cells were seeded in six-well plates overnight and co-transfected with the indicated plasmids. At 24 h, cells were infected with GCRV (multiplicity of infection [MOI] = 10) for 48 h, and the cell lysates were subjected to IB with the anti-STING, anti-Flag, and anti-*β-actin* Abs, respectively. (**H–P**) Overexpression of TOLLIP promotes the inhibition of STING-induced IFN activation by VP4, whereas knockdown of *tollip* reversed this. GCO cells were seeded in 24-well plates overnight and transfected with 250 ng IFN1pro-Luc or ISRE-Luc, 250 ng TOLLIP-Myc or empty vector, 250 ng STING-HA (**H and I**), 250 ng sh-*tollip*#2 (**M and N**), or empty vector and 50 ng pRL-TK, together with 180 ng VP4-Flag or empty vector. At 24 h, cells were lysed for luciferase activity detection. The promoter activity is presented as RLUs normalized to *Renilla* luciferase activity. GCO cells seeded in six-well plates overnight were transfected with indicated plasmids. At 24 h, total RNAs were extracted to examine the mRNA levels of cellular *ifn* (**O**) and *isg15* (**P**). The relative transcriptional levels were normalized to the transcription of *β-actin* and represented as fold induction relative to the transcriptional level in the control cells, which was set to 1. Data were expressed as mean ± SEM, *n* = 3. The *P* values were calculated by one-way analysis of variance with Dunnett’s post hoc test (SPSS Statistics, version 19; IBM). Asterisks indicate significant differences from the control (*P < 0.05). All experiments were repeated for at least three times with similar results.

Autophagy can selectively degrade target molecules by using autophagy receptors that bind to autophagosomes and recruit substrates (31). To further explore the precise mechanism of VP4-mediated autophagic degradation of STING, several autophagy-selective receptors were cloned and screened. In Co-IP assays, it was found that HA-tagged VP4 specifically interacts with Flag-tagged TOLLIP/Parkin in GCO cells (Fig. 6A). The results of the reverse IP assays demonstrated a specific association between VP4 and TOLLIP while excluding the interaction between VP4 and Parkin (Fig. 6B and C). Meanwhile, the Co-IP assays also revealed that TOLLIP was associated with STING (Fig. 6D and E). Confocal microscopy analysis showed patchy localization of TOLLIP’s green signals and STING’s or VP4’s red signals in the cytoplasm (Fig. 6F). Therefore, we hypothesized that TOLLIP plays a role in VP4-mediated autophagic degradation of STING. As anticipated, overexpression of TOLLIP significantly enhanced the degradation of STING by VP4 (Fig. 6G). Moreover, the inhibition of STING-induced activities of IFN1pro and ISRE by VP4 was more pronounced in the TOLLIP-overexpressed group (Fig. 6H and I). Next, we designed two short hairpin RNAs (shRNAs) targeting TOLLIP for subsequent assays, and sh-*tollip*#2 was found to be more effective (Fig. 6J). In addition, *tollip* knockdown also dramatically blocked the degradation of the endogenous and exogenous of STING by VP4 (Fig. 6K and L). Furthermore, knockdown of *tollip* reversed the inhibition of STING-activated IFN1pro and ISRE activity by VP4 (Fig. 6M and N). At the mRNA level, the inhibition of STING-induced expression of *ifn* and *isg15* by VP4 was reversed by sh*-tollip*#2 (Fig. 6O and P). Taken together, these results suggest that VP4 utilizes TOLLIP to degrade STING in the autophagy-lysosome pathway.

### VP4 plays a vital role in GCRV’s proliferation

**Fig 7:**
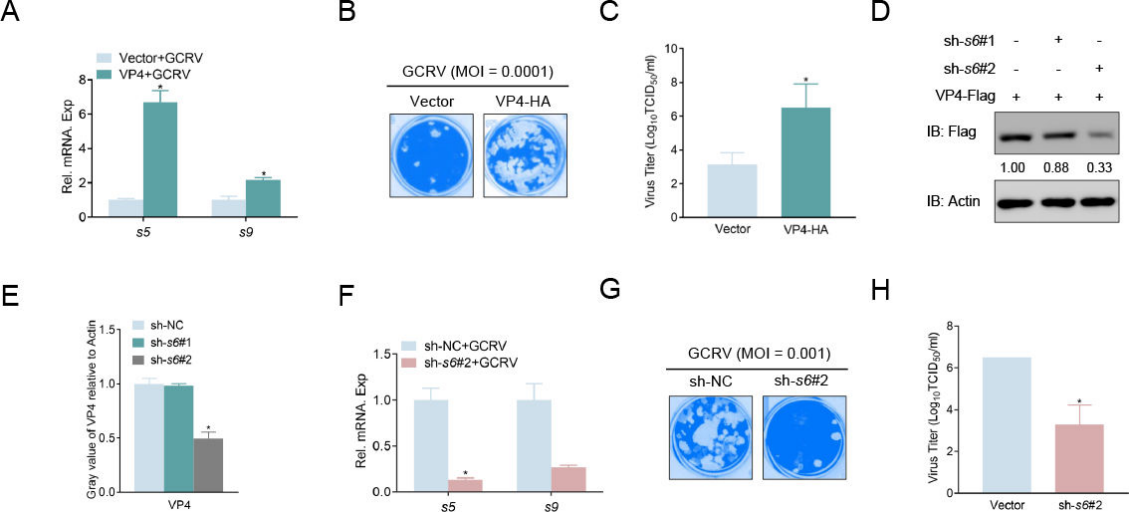
VP4 inhibits the cellular IFN response and facilitates viral RNA synthesis. (**A and F**) VP4 promotes the proliferation of viral genes. GCO cells seeded in six-well plates were transfected with indicated plasmids (2 µg each). At 24 h, cells were infected with GCRV (multiplicity of infection [MOI] = 10). After 24 h, total RNAs were extracted to examine the mRNA levels of *s5* and *s9*. The relative transcriptional levels were normalized to the transcription of *β-actin* and represented as fold induction relative to the transcriptional level in the control cells, which was set to 1. Data were expressed as mean ± SEM, *n* = 3. Asterisks indicate significant differences from the control (*P < 0.05). (B, C, G, and H) GCO cells seeded in 24-well plates overnight were transfected with indicated plasmids (0.5 µg each). At 24 h, cells were infected with GCRV (MOI = 0.0001 or 0.001) for 48 h. After 48 h, the supernatant was transferred to in CIK cells seeded in 24-well for 48 h. Then, cells were fixed with 4% PFA and stained with 1% crystal violet. Culture supernatants from the CIK cells infected with GCRV were collected, and the viral titer was measured according to the method of Reed and Muench. (**D and E**) Validation of knock-down efficiency of VP4. GCO cells were seeded in six-well plates overnight and transfected with 1 µg VP4-Flag and 1 µg sh-*s6*#1-PLKO.1, sh-*s6*#2-PLKO.1, or control (sh-NC). At 24 h, the cell lysates were subjected to immunoblotting (IB) with the anti-Flag and anti-*β-actin* Abs. All experiments were repeated at least three times with similar results.

To determine whether VP4 affects GCRV amplification, GCO cells were transfected with VP4-HA and stimulated with GCRV. The quantitative PCR (qPCR) assays showed upregulation of *s5* and *s9* of GCRV in the VP4-overexpressed group compared to the control group (Fig. 7A). Additionally, more CPEs were observed, and viral titers increased 2,344-fold (from 10^3.13^ to 10^6.50^ TCID_50_/mL at 48 h PI) in the VP4-overexpressed cells (Fig. 7B and C). In addition, two shRNAs targeting VP4 were designed based on the nucleic acid sequence of *s6*. Among them, sh*-s6#2* was found to be effective (Fig. 7D and E). In contrast, the knockdown of *s6* suppressed the expression of GCRV *s5* and *s9* at the mRNA level (Fig. 7F). Meanwhile, *s6* knockdown caused a decrease in CPE and a 1,622-fold decline in viral titer (from 10^6.50^ to 10^3.29^ TCID_50_/mL at 48 h PI; Fig. 7G and H). These results indicate that the overexpression of VP4 promotes GCRV proliferation and vice versa.

## DISCUSSION

Fish possess a potent IFN immune system to protect against viral infections. However, GCRV can still penetrate the host immune response, resulting in the death of many farmed fish and causing huge economic losses in the aquaculture industry. Limited research has been conducted on the immune escape mechanism of GCRV. In this study, we showed that the GCRV VP4 interacts with STING, leading to the autophagic degradation of STING to inhibit IFN production. In summary, the findings reveal a novel immune escape strategy employed by GCRV, which enhances the understanding of viral pathogenesis.

Currently, the immune escape mechanisms for GCRV are more specific to GCRV-II. For instance, VP41 and VP56 of GCRV-II block IFN expression by attenuating STING phosphorylation or degrading IRF7 through the proteasome pathway, respectively (32, 33). Additionally, it has been reported that GCRV-II inhibits TBK1 activation by preventing K63-linked ubiquitination and promoting K48-linked ubiquitination of TBK1, thereby evading IRF7-mediated antiviral immune responses (34). In general, GCRV-II evades host immune response through autophagic degradation, ubiquitin-proteasome degradation, and phosphorylation competition inhibition. The immune escape strategies of GCRV-I are less well studied. In this study, we found that VP4 of GCRV-I degrades STING through the autophagy pathway, which differs from the escape strategy of VP4 in GCRV-II studies. It is confirmed that various types of GCRVs have distinct functions for their encoded proteins. Further exploration is required to understand other immune escape tactics employed by GCRV-I.

The existing research on VP4 proteins from other reoviruses is limited, with most studies concentrating on GCRV-II. For instance, Su et al. showed that VP4 of GCRV-097 interacts with RIG-I to inhibit the IFN response and facilitate GCRV invasion (35). Furthermore, VP56 of GCRV-097 binds to VP4 in the cytoplasm, enhancing the degradation of RIG-I and promoting viral replication more effectively (36). In our study, we present for the first time the role of the VP4 protein in GCRV-I, emphasizing that the degradation of STING by VP4 is crucial for GCRV immune evasion. Additionally, we compared the VP4 protein sequence of GCRV-873 with other reoviruses protein sequences exhibiting over 50% identity. Most of these viral proteins lacked any documented function. Only VP5 of GCRV-096 is predicted to facilitate GCRV entry into host cells, while VP4 of HGCRV is thought to have a conserved structural domain derived from the heavy viral_M2 superfamily of proteins that affect host cell membrane permeability (37, 38). It is not yet established whether other reoviruses possess VP4-like functionality. Furthermore, it is necessary to confirm whether the VP4 knockdown observed in Fig. 7 resulted in viral clearance due to the inability to inhibit IFN expression or because VP4 acted as a vesicle membrane protein of GCRV to impede viral replication. It would indeed be more beneficial to demonstrate the consistency of VP4 in immune evasion and viral replication through cellular experiments in actual organisms. Thus, additional studies will be conducted to confirm the role of VP4 in the *in vivo* infection model by utilizing mutant strains of the virus.

STING is an important adaptor protein responsible for the RLR pathway and plays a vital role in RNA- and DNA-triggered IFN immune responses in mammals (24, 39). Similarly, fish STING also displays a powerful antiviral function by inducing IFN expression (24, 25). Therefore, many viruses attempt to intercept STING signaling through a variety of strategies, including interaction disruption, cleavage, and post-translational modifications (40–42). For example, the hemagglutinin fusion peptide (FP) of influenza virus A interacts with STING and eliminates STING-dependent IFN induction by membrane fusion (43). 2a NS4B of HCV inhibits STING accumulation, and NS2B3 of dengue virus cleaves STING directly, thereby inhibiting IFN production (40, 44). VP1-2 of herpes simplex virus type 1 and Tax of human T lymphotropic virus type 1 decreased the K63-linked ubiquitination of STING and block IFN expression (45, 46). Fish STING have a conserved structure among vertebrates, which includes four N-terminal TMs that determine its localization to the ER and a cytosolic C-terminal domain. Recent studies have shown that viruses can target STING by disrupting their translocation to the Golgi apparatus or perinuclear microsomes (47–49). In this study, it was found that VP4 of GCRV interacts with STING via the TM region, which is necessary for STING to localize to the ER. Whether VP4 affects STING’s translocation to evade STING’s signal transmission deserves to be determined.

Autophagy is a homeostatic process in eukaryotic cells that sequesters intracellular components into autophagosomes, then fuse with lysosomes to degrade the captured substrate for recycling (50). Recent studies have identified that autophagy can be classified into nonselective and selective autophagy. The substrates in selective autophagy processes are engulfed into autophagosomes in a selective manner. Autophagy cargo receptors such as SQSTM1/p62 (sequestosome 1), NBR1 (NBR1 autophagy cargo receptor), OPTN (optineurin), CALCOCO2/NDP52 (calcium binding and coiled-coil domain 2), BNIP3L/Nix (BCL2 interacting protein 3 like), and TOLLIP mediate these processes (51, 52). Among them, TOLLIP is utilized by many viruses to eliminate cellular protein aggregates. For instance, Jin et al. discovered that SUMOylation restrains the degradation of the angiotensin-converting enzyme 2 receptor through TOLLIP-mediated selective autophagy, thereby increasing host susceptibility to SARS-CoV-2 (53). Ma et al. found that the tegument protein UL21 of pseudorabies virus (PRV) inhibits innate immunity by triggering cGAS degradation through TOLLIP-mediated selective autophagy (54). Cheng et al. found that L83L of ASFV interacts with cGAS and STING, recruiting TOLLIP to promote autophagy-lysosomal degradation of STING, eventually reducing IFN-I signaling (55). Additionally, Yan et al. found that the PRV UL38 diminishes the cGAS-STING signaling pathway by recruiting TOLLIP to promote the autophagic degradation of STING (56). The above-mentioned studies on the relationship between TOLLIP and STING have primarily concentrated on dsDNA viruses. Our results examined the relationship between TOLLIP and STING in RNA viruses. This means that most viruses may be able to use TOLLIP to attack STING.

Furthermore, our study examines the role of TOLLIP in the immune escape mechanism of GCRV. The study found that TOLLIP interacts with STING and is essential for VP4-mediated autophagic degradation of STING. These degradations were alleviated after *tollip* knockdown. However, the selective interaction of TOLLIP with STING and its regulation of autophagosome formation necessitates further mechanistic exploration. Additionally, whether VP4 induces ubiquitination modification of STING in this process, allowing it to be recognized by TOLLIP, warrants further investigation.

In conclusion, the study presented a novel role for GCRV VP4 in recruiting TOLLIP to negatively regulate IFN responses by degrading STING via the autophagy-lysosome pathway. These findings offer valuable insights into GCRV immune evasion mechanisms and can aid in the prevention and control of aquatic diseases.

## MATERIALS AND METHODS

### Cells and viruses

GCO cells and CIK cells were obtained from China Center for Type Culture Collection and were maintained at 28°C in 5% CO_2_ in medium 199 (M199; Invitrogen) supplemented with 10% fetal bovine serum. GCRV (strain 873, group I) was provided by Prof. Wuhan Xiao (Institute of Hydrobiology, Chinese Academy of Sciences). GCRV was propagated in CIK cells until a CPE was observed. Then, the harvested cell culture fluid containing GCRV was centrifuged at 4,000 × *g* for 20 min to remove the cell debris, and the supernatant was stored at −80°C until used.

### Plasmid construction and reagents

The sequence of *s6* (GenBank accession number: AF403392.1) that encodes VP4 was obtained from the NCBI (National Center for Biotechnology Information) website (http://www.ncbi.nlm.nih.gov/). Using the cDNA of the cell lysate from GCRV-infected CIK cells as a template, the ORF of VP4 was amplified by PCR and cloned into pCMV-Flag, pCMV-Myc, and pcDNA3.1(+) vectors (Clontech, Mountain View CA, U.S.A), respectively. The ORFs of MAVS (KF366908.1), STING (JN786909.1), TBK1 (JN704345.1), IRF3 (KT347289.1), and IRF7 (KY613780.1) cloned into pCMV-Flag and pCMV-Myc vectors (Clontech, Mountain View CA, U.S.A). OPTN (XM_051892472.1), p62 (MN311522.1), parkin (XM_051917228.1), and TOLLIP (XM_051899887.1) were also subcloned into pCMV-Flag vector. For subcellular localization, the ORF of VP4 was inserted into the pEGFP-N3 or pDsRed-N1 vector (Clontech). The ORFs of TOLLIP were inserted into the pCS2-mCherry (Clontech) and pEGFP-N3 vector. The ORF of STING and the truncated mutants of STING were also subcloned into the pCS2-mCherry vector. The ORFs of microtubule-associated protein 1 light chain 3 (LC3, MG821471.1) were inserted into the pEGFP-N3 vector. For promoter activity analysis, IFN1pro-Luc construct was generated by insertion of corresponding 5′-flanking regulatory region of IFN1 promoter (GU139255.1) into pGL3-basic luciferase reporter vector (Promega, Madison, WI). The ISRE-Luc plasmid in the pGL3-basic luciferase reporter vector (Promega) was constructed as described previously (57). The *Renilla* luciferase internal control vector (pRL-TK) was purchased from Promega. All constructs were confirmed by DNA sequencing.

### Transient transfection and virus infection

Transient transfections were performed in GCO cells seeded in 6-well or 24-well plates by using FishTrans (MeiSenTe Biotechnology, Guangdong, China). For the antiviral assay using 24-well plates, GCO cells were transfected with 0.5 µg VP4-Flag or the empty vector for 24 h, and then GCRV at a multiplicity of infection (MOI) of 0.001 was added. After 48 h, the supernatant was transferred to in CIK cells seeded in 24-well. Antiviral assays were implemented as described (58). For the qPCR assay, GCO cells were seeded in six-well plates and transfected with 2 µg VP4-Flag or the empty vector. After 24 h, the cells were transfected with poly I:C or infected with GCRV (MOI = 10) for 24 h, and total RNAs were extracted to examine the mRNA levels.

### RNA interference

The shRNA of *s6* and *tollip* was designed by BLOCK-iT RNA interference (RNAi) Designer and cloned into the pLKO.1-TRC cloning vector. For RNAi of *s6*, GCO cells were seeded in six-well plates overnight and transfected with 2 µg shRNA of *s6* or the negative control (sh-NC) for 24 h, and then GCRV (MOI = 0.001) was added. After 24 h, cells were harvested for RNAi effectiveness detection. For RNAi of *tollip*, GCO cells were seeded in six-well plates overnight and transfected with 2 µg shRNA of *tollip* or the negative control (sh-NC) and TOLLIP-Flag for 24 h. After 24 h, cells were harvested for RNAi effectiveness detection. The following sequences were targeted for *s6* and *tollip*: sh-*s6*#1 (GCAAAGACCTCGATCTAATTG), sh-*s6*#2 (GCGGAATTCAATAACTCATCC), sh-*tollip*#1 (GCAACAGCAGGTACAGTTAGA), and sh-*tollip*#2 (GGACTCCTTCTACCTGGAAAT).

### Luciferase activity assay

GCO cells were seeded into 24-well plates overnight and co-transfected with various constructs at a ratio of 5:5:5:1 (VP4, STING, IFN 1pro/ISRE-Luc, and pRL-TK expression vectors). Transfection of poly I:C was performed 24 h before cell harvest. At 48 h post-transfection, luciferase activity was measured with the Dual-Luciferase Reporter Assay system (Promega) according to the manufacturer’s instructions. Firefly luciferase activities were normalized based on *Renilla* luciferase activity. The results are representative of data from more than three independent experiments, each performed in triplicate.

### RNA extraction, reverse transcription, and qPCR

Total RNAs were extracted using the Trizol reagent (Invitrogen). cDNA was synthesized using PrimeScript reverse transcription reagent kit (TaKaRa, Kyoto, Japan), according to the manufacturer’s instructions. qPCR was performed with Fast SYBR Green PCR Master Mix (Bio-Rad) on the CFX96 Real-Time System (Bio-Rad). PCR conditions were as follows: 95°C for 5 min and then 40 cycles of 95°C for 20 s, 60°C for 20 s, and 72°C for 20 s. The *β-actin* gene was used as an internal control. The relative fold changes or relative mRNA of level were calculated by comparison to the corresponding controls using the 2^−ΔΔCT^ method. Three independent experiments were conducted for statistical analysis. The following qPCR primer sequences: *ifn*, 5′-CCGATACCAGCCATCACATAAG-3′ and 5′-GATCTGCTCCCATGCTTGAG-3′; *isg15*, 5′-GGTGAAAGTTGATGCCACAGTTG-3′ and 5′-TTGGAAAGGGGGGTTCGTG-3′; *vig1*, 5′-TTCCACACTGCGAAGACCTC-3′ and 5′-CCATTACTAACGATGCTGACGC-3′; *sting*, 5′-CTCATGGTTTGGCCTGGTCT-3′ and 5′-GTTTGTTTGGGACCACAGCG-3′; *β-actin*, 5′-AGCCATCCTTCTTGGGTATG-3′ and 5′-GGTGGGGCGATGATCTTGAT-3′; *s5*, 5′-CCCTTACCGCTTCTGAACT-3′ and 5′-GGATGCTTGGACGCTACA-3′; *s6*, 5′-GTGTTGACCCTGGATGTGAG-3′ and 5′-GTTAGCAGCGGTAGTGACTTG-3′; *s8*, 5′-CCCTGACTGGACGCCTAA-3′ and 5′-CGCCTGCCACTTCTACGA-3′; *s9*, 5′-GCCGCTCGTGATTTGTTA-3′ and 5′-GGGTAGGTGTCGGGTAGTTC-3′.

### Co-IP assay

For Co-IP experiments, GCO cells seeded in 10 cm^2^ dishes overnight were transfected with 5 µg each plasmid indicated on the figures. At 24 h post-transfection, the medium was removed carefully, and the cell monolayer was washed twice with 10 mL ice-cold PBS. Then the cells were lysed in 1 mL of radioimmunoprecipitation lysis buffer (1% NP-40, 50 mmol/L Tris-HCl, pH 7.5, 150 mmol/L NaCl, 1 mmol/L EDTA, 1 mmol/L NaF, 1 mmol/L sodium orthovanadate [Na_3_VO_4_], 1 mmol/L phenylmethylsulfonyl fluoride, and 0.25% sodium deoxycholate) containing protease inhibitor cocktail (Sigma-Aldrich) at 4°C for 1 h on a rocker platform. The cellular debris was removed by centrifugation at 12,000 × *g* for 15 min at 4°C. The supernatant was transferred to a fresh tube and incubated with 30 µL anti-Flag/HA/Myc affinity gel (Sigma-Aldrich) overnight at 4°C with constant rotating incubation. These samples were further analyzed by immunoblotting (IB). Immunoprecipitated proteins were collected by centrifugation at 5,000 × *g* for 1 min at 4°C, washed for three times with lysis buffer, and resuspended in 100 µL 1 × SDS sample buffer. The immunoprecipitates and whole cell lysates (WCLs) were analyzed by IB with the indicated antibodies (Abs).

### Immunoblot analysis

Immunoprecipitates or WCLs were analyzed as described (58, 59). Abs were diluted as follows: anti-*β-actin* (ABclonal, AC026) at 1:3,000, anti-Flag (Sigma-Aldrich, F1804) at 1:3,000, anti-HA (Covance, MMS-101R) at 1:3,000, anti-Myc (Santa Cruz Biotechnology, sc-40) at 1:3,000, and anti-LC3 (Abcam, ab48394) at 1:1,000. The indicated Ab of STING proteins at 1:2,000 was prepared by our lab.

### Transmission electron microscopy

GCO cells were seeded in six-well plates and transfected with indicated plasmids using FishTrans (MeiSenTe Biotechnology) for 24 h. For pretreatment, cells were washed with PBS, trypsinized, and transferred to 1.5 mL centrifuge tube. Cells were pelleted by centrifugation at 2,000 × *g* for 5 min. The cell pellets were resuspended with 2.5% glutaraldehyde in 0.075 mol/L phosphate buffer (pH 7.4) for 4 h at 4°C for prefixation. Then the cells were washed for three times with a solution containing 0.075 mol/L phosphate and 0.19 mol/L sucrose for 15 min each and post-fixed in 1% osmium tetroxide (OsO_4_) in 0.24 mol/L phosphate buffer (pH 7.4) for 2 h. After being washed for three times for 15 min each in 0.075 mol/L phosphate buffer and 0.19 mol/L sucrose buffer at 4°C, the cells were dehydrated with a graded series of ethanol and acetone and then gradually infiltrated with epoxy resin. Samples were sequentially polymerized at 37°C overnight and then 60°C for 48 h. Ultrathin sections (74 nm) were cut using microtome (UC7; Leica) and mounted on copper slot grids. Sections were doubly stained with 3% uranyl acetate-lead citrate for 10 min and observed under transmission electron microscope (HT7700; Hitachi, Tokyo, Japan).

### Fluorescent microscopy

Fluorescence confocal detection of GCO cells was generated as described (59).

### Statistics analysis

qPCR data analyzed for significance were performed as mean SEM. The *P* values were calculated using the student’s *t*-test or one-way analysis of variance with Dunnett’s post hoc test (SPSS Statistics, version 19; IBM). A *P* value < 0.05 was considered statistically significant.

## Data Availability

The authors confirm that the data supporting the findings of this study are available within the article.
